# Burst Phase Analysis of the Aggregation Prone α-synuclein Amyloid Protein

**DOI:** 10.1007/s10895-023-03285-1

**Published:** 2023-06-05

**Authors:** Marco A. Saraiva, M. Helena Florêncio

**Affiliations:** 1 Centro de Química Estrutural, Institute of Molecular Sciences, Av. Rovisco Pais, Instituto Superior Técnico, University of Lisbon, Campus Alameda, Lisbon, 1049-001 Portugal; 2 Departamento de Química e Bioquímica, Faculdade de Ciências, University of Lisbon, Lisbon, 1749- 016 Portugal; 3 Laboratório de FTICR e Espectrometria de Massa Estrutural, Faculdade de Ciências, University of Lisbon, Lisbon, 1749-016 Portugal; 4 MARE – Marine and Environmental Sciences Centre / ARNET - Aquatic Research Network, Faculdade de Ciências, University of Lisbon, Lisbon, 1749-016 Portugal

**Keywords:** α-synuclein, Stopped-flow, Protein aggregation, Burst phase analysis, Fluorescence spectroscopy

## Abstract

While some studies inferred that valid information can be retrieved for the refolding of proteins and consequent identification of folding intermediates in the stopped-flow spectrometry collapse phase, other studies report that these burst phase folding intermediates can be questioned, implying a solvent-dependent modification of the still unfolded polypeptide chain. We therefore decided to investigate the burst phase occurring for the α-synuclein (Syn) amyloid protein by stopped-flow spectrometry. Solvent-dependent modification effects indeed occurred for the *N*_α_-acetyl-L-tyrosinamide (NAYA) parent small compound and for the folded monomeric ubiquitin protein. More complex was the burst phase analysis of the disordered Syn amyloid protein. While this amyloid protein was determined to be aggregated at pH 7 and pH 2, in particular, this protein at pH 3 appears to be in a monomeric state in the burst phase analysis performed. In addition, the protein at pH 3 appears to suffer a hydrophobic collapse with the formation of a possible folded intermediate. This folded intermediate seems to result from a fast contraction of the disordered amyloid polypeptide chain, which is proceeded by an expansion of the protein, due to the occurrence of solvent-dependent modification effects in a milliseconds time scale of the burst phase. Generally, it can be argued that both literature criteria of solvent-dependent modifications of the disordered Syn amyloid protein and of the formation of its possible folded intermediate are very likely to occur in the burst phase.

**Supplementary Information:**

The online version contains supplementary material available at 10.1007/s10895-023-03285-1.

## Introduction

In some cases, the existence of protein intermediates can be inferred from the effect of denaturants on the kinetics of protein folding [1]. In contrast to the V-shape behaviour, expected for simple two-phase folding/unfolding transition, the rate profile for many proteins exhibit the linear two-state behaviour only in the unfolding-transition region, but deviates markedly at low denaturant concentration where the observed rate of folding levels off [1]. In the initial studies on the barnase protein, the authors attributed this effect to an early folding intermediate formed during the mixing time [2]. Other studies have shown that when some unfolded proteins are diluted from high denaturant in folding experiments, they experience a fast polymer chain contraction from a more extended form of the unfolded state, to a more contracted form of the unfolded state [3]. The optical signals produced by the chain contraction can misleadingly mimic the formation of a distinct intermediate [3]. Nevertheless, the suppose intermediate is the unfolded state itself [3]. In other words, when proteins are diluted from concentrated denaturant at the beginning of a stopped-flow folding experiment, they very generally exhibit a fast chain contraction, on a sub millisecond time scale, that can be detected by means of various probes [4]. This ‘burst phase’ behavior has often been interpreted in terms of the fast formation of productive folding intermediates [4]. But, for example, some studies performed on truncated cytochrome *c* variants that cannot fold produce the same burst phase fluorescence and circular dichroism (CD) as the intact protein [4]. Therefore, this aspect suggests that the burst phase does not reflect the formation of a distinct folding intermediate, but rather some solvent-dependent modification of the still unfolded polypeptide chain [4].

To this report, we investigated the two above literature criteria of the solvent-dependent modification effects and of the formation of a folded intermediate by fast chain contraction for the disordered α-synuclein (Syn) amyloid protein in the burst phase [3, 4]. For the NAYA small parent compound and for the folded ubiquitin (UBQ) protein at pH 7, we have determined that solvent-dependent modification effects occurred in the burst phase. For the disordered Syn protein, at pH 3, in particular, determined to be in a monomeric state [5], we inferred that there is a fast chain contraction of the disordered protein leading to the formation of a possible folded intermediate in the millisecond time scale of the burst phase. In addition, we, therefore, assigned the solvent-dependent modification effects as a possible contributor to the first stage of the above process, leading to the referred fast chain contraction of the disordered Syn protein, which is the amyloid protein expansion.

## Materials and Methods

### Materials

The *N*_α_-acetyl-L-tyrosinamide (NAYA) and the bovine ubiquitin protein (UBQ) (≥ 98%, by SDS-PAGE) were purchased from Sigma-Aldrich and used without further purification. The 8-hydroxyquinoline compound was from Merck. As for the magnesium chloride 6-hydrate, it was from Panreac.

### Syn Expression and Purification

The pT7-7 plasmid containing the human Syn sequence (kindly provided by Professor Doctor T. Outeiro, IMM, University of Lisbon) was used to overexpress Syn in Escherichia coli BL21 (DE3) bacteria. Syn was purified as previously described [6–13].

### SFM-4 Stopped-Flow Experiments

For the earliest 40 milliseconds, stopped-flow measurements were performed in a Biologic SFM-4 apparatus connected, via an optic fibber, to a xenon-mercury lamp, and equipped with a circulating water bath for temperature control (experiments were carried out at 20.0 ºC) [6]. The SFM module is equipped with a specially designed mixer (HDS) that includes an internal siphon-like frame and allows blockage of convection created by density or temperature differences [11]. Moreover, in the fluorescence intensity measurements, the tyrosine excitation was at 275 nm and the fluorescence emission was collected above 290 nm, using a 290 nm Schott cut-off filter [6]. Also, in the light scattering measurements, the excitation was at 378 nm and the light scattered was recorded above 290 nm, using the same 290 nm Schott cut-off filter [6]. For pH-jumps, the experiments were carried out by mixing the molecular species solutions in 10 mM tris-HCl at pH 7 with (HCl) acidified buffer to give the final desired pH. In order to vary the molecular species concentration (10 mM tris-HCl), the experiments were performed by automatically changing the ratio of the stopped-flow syringes used. It deserves to be mentioned that in these latter experiments the flow rate varied automatically between 8 mL/s to 6.5 mL/s in the measurements, i.e. depending on the relative and total volumes used.

## Results

### Burst Phase Analysis for the NAYA Parent Compound

**Fig. 1:**
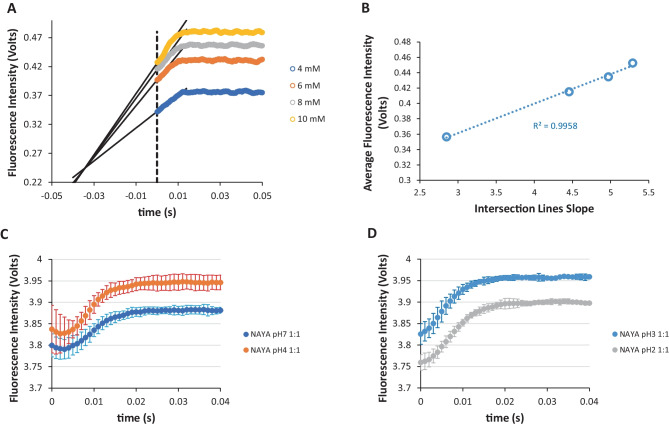
**A** Determination of the zero time of the SFM-4 stopped-flow instrument using the 8-hydroxyquinoline-Mg^2+^ complex, in the fluorescence detection mode. By varying the 8-hydroxyquinoline compound concentration the fluorescence intensity curves intersected at ca. -44 milliseconds. **B** Average values of the 8-hydroxyquinoline-Mg^2+^ complex fluorescence intensity (first 100 milliseconds) as a function of the intersection lines slope. In this representation, a linear dependence was observed. SFM-4 stopped-flow fluorescence intensity traces for the NAYA compound as function of the solution pH (first 40 milliseconds). **C** NAYA solutions at pH 7 and pH 4 and **D** NAYA solutions at pH 3 and pH 2. The data in this figure is from two independent experiments, i.e. the data was recorded in different days. Error bars correspond to the standard deviation of three reproducible measurements (*N* = 3 ± std) for each independent experiment

Burst phase analysis is a complex experiment. While some studies inferred that valid information can be retrieved for the refolding of proteins and consequent identification of folded intermediates in the collapse phase [1, 2], other studies report that these burst phase folded intermediates can be questioned implying a solvent-dependent modification of the still unfolded polypeptide chain [3, 4]. We decided therefore to investigate the burst phase occurring for the Syn amyloid protein by stopped-flow spectrometry. In spite of treating this subject carefully, as it is rather controversial in the literature, we decided to firstly investigate the possible burst phase occurrence for the NAYA parent small compound. This compound possesses in its molecular structure a tyrosyl group, similarly to the four tyrosyl groups present in the Syn protein molecular structure and, moreover, its mimetics the peptide bonds encountered in proteins. The SFM-4 stopped-flow instrument used in the rapid mixing experiments has a particular feature that enables the acquisition of data before the stop. To this purpose, we decided, by default conditions, to monitor the 10 milliseconds before the stop. The zero time (different from the dead time) corresponds to the start of the resulting rapid mixing of the starting solutions, which eventually stops, in the stopped-flow observation cell. In order to validate our results, we firstly decided to determine the zero time of the SFM-4 stopped-flow instrument, in an attempt to be assured that the above referred default conditions, of the recording 10 milliseconds before the stop, were actually reporting to the situation that rapid mixing has already started. In Fig. 1A, we used the 8-hydroxyquinoline-Mg^2+^ complex to verify the above situation, in the fluorescence detection mode [14]. Therefore, by varying the 8-hydroxyquinoline compound concentration, the obtained stopped-flow traces intersected at the time of ca. -34 milliseconds, before the acquisition started, by default acquisition conditions of 10 milliseconds, before the stop. To this regard, we are in position to infer that the default acquisition conditions of 10 milliseconds before the stop in fact reported for the event of rapid mixing of the starting solutions. Also, the referred zero time in the SFM-4 stopped-flow experiments is ca. -44 milliseconds, of which 10 milliseconds actually report for the default acquisition conditions, used before the stop. We are confident of this zero time value determined since we plotted the average values of the 8-hydroxyquinoline-Mg^2+^ complex fluorescence intensity (first 100 milliseconds) as a function of the slope of the intersection lines and a linear dependence was observed (Fig. 1B).

With respect to the NAYA parent compound, we present in Fig. 1C, D the stopped-flow traces obtained for a final 1.34 × 10^− 4^ M concentration (*A*_275 nm_ = 0.2; ε = 1390 M^− 1^ cm^− 1^). A solution containing the NAYA parent compound in buffer (10 mM tris-HCl) was mixed in an equal volume, with the same buffer solution without this compound. The pH of the resulting solutions was also varied in the buffer solutions without the NAYA compound. The final pH of the NAYA compound solutions investigated were pH 7, 4, 3 and 2. Still, in Fig. 1C, D, can clearly be observed that a fluorescence intensity signal perturbation occurs in the first 20 milliseconds, of which the first 10 milliseconds retrieve to the default acquisition conditions used before the stop. After the referred 20 milliseconds of data acquisition, the stopped-flow traces entered in a stationary phase, i.e. what it can be called by the steady-state of the fluorescence intensity measurements. No particular reaction is to be expected for the NAYA parent compound while varying the solution pH from 7 to 2. And, the differences in the recorded fluorescence intensity signal in the steady-state are certainly attributed to an expected fluorescence intensity signal fluctuation in the measurements, i.e. (0.1 V / 3.9 V) × 100 (%) = 2.6% error. The referred first 20 milliseconds of the stopped-flow traces, before entering in a stationary phase, are due to the occurrence of the burst phase. A close inspection of Fig. 1D reveals that for NAYA solutions at pH 3 and pH 2 there is mostly a rise of the fluorescence intensity in the burst phase, while for NAYA solutions at pH 7 and pH 4 the scenario is different (Fig. 1C). In this latter situation, there is initially a decrease of the recorded fluorescence intensity, i.e. in the first 3 milliseconds, followed by a rise of the fluorescence intensity in the remaining 17 milliseconds of the burst phase (Fig. 1C). However, there are questions that should be answered. Firstly, what is the reason for the variation of the fluorescence intensity of NAYA solutions in the burst phase, since no particular reaction is expected to occur? Secondly, why are there differences in the recorded fluorescence intensity for the NAYA solutions while varying the solution pH? These questions recall to the situation where a solvent-dependent modification can occur in the NAYA solutions and this seems to be itself a proof for the occurrence of burst phase in the stopped-flow experiments. We thought about an explanation for this, in order to answer for the above two questions and, such an explanation can be given in the light of the classic rotameric conformations of the NAYA parent compound [15]. In two recent studies, we have investigated the exposure of the NAYA tyrosyl group in aqueous solution [11, 13]. In one study, we added the non-polar 1,4-dioxane solvent to the NAYA aqueous solutions [11] and, in another study, we investigated the effect to the initial temperature rise of NAYA aqueous solutions [13]. In these two studies we use the classic rotameric conformations of the NAYA parent compound [15] in order to interpret the referred likely exposure of the NAYA tyrosyl group in aqueous solution, as a strategy that, was adopted to understand the aggregation behavior of the Syn amyloid protein under similar solution conditions [11, 13]. As above referred, there is initially a decrease of the recorded fluorescence intensity, i.e. in the first 3 milliseconds, for the NAYA pH 7 and pH 4 solutions (Fig. 1C). If the burst phase is occurring in NAYA stopped-flow experiments, it is expected that hydrophobic groups become exposed to water [16], such as the NAYA tyrosyl group. Therefore, the initial decrease of the collected fluorescence intensity in the first 3 milliseconds, for NAYA pH 7 and pH 4 solutions, can be due to the progressive quenching of the NAYA tyrosyl group fluorescence intensity, as the turbulence of the stopped-flow solutions become minor. As it is expected, there is no turbulence when the NAYA solutions are lately in the stationary phase. Concerning the NAYA rotameric conformations [15], the *gauche*(+) rotamer can represent the fully water exposed NAYA tyrosyl group when both NAYA amide carbonyl and NAYA acetyl carbonyl can be far apart from the NAYA tyrosyl group, specifically in a closed shape conformation [17]. And this locked *gauche*(+) rotamer structure can be the most populated in the NAYA solutions in the first 3 milliseconds of the stopped-flow curves depicted in Fig. 1C. The other two NAYA rotameric conformations *trans* and *gauche*(−), in which there is interaction with the NAYA amide carbonyl and NAYA acetyl carbonyl with the NAYA tyrosyl group, respectively, can be the most populated NAYA rotameric conformations in the last 17 milliseconds of the burst phase. This is due to the referred hydrophobic interactions of the NAYA carbonyl groups with the NAYA tyrosyl group that are responsible for the progressive increase of the NAYA fluorescence intensity as the turbulence of the NAYA solutions becomes minor with the time of the burst phase. Overall, the NAYA tyrosyl group exposed to solvent molecules in the locked *gauche*(+) rotamer structure suffers, in the burst phase, increased fluorescence intensity quenching in comparison with that for the less exposed NAYA tyrosyl group in interaction with the NAYA carbonyl groups. Therefore, the predominance of the fluorescence intensity quenching for the locked NAYA *gauche*(+) rotamer is expected in beginning of the burst phase, as well as an increase of the fluorescence intensity for the NAYA *trans* and *gauche*(−) rotamers at the end of the burst phase. The result is the NAYA rotamer populations being differentiated in the burst phase time, due to the fact that the NAYA tyrosyl group exposure in the burst phase is distinct from the one in the stationary phase (quiescent conditions). With respect to the abolishment of the 3 milliseconds event in the NAYA solutions at pH 3 and pH 2, in comparison to NAYA solutions at pH 7 and pH 4, we can infer that the most populated locked NAYA *gauche*(+) rotamer structure is rather influenced in this case by the increased concentration of protons. In the locked NAYA *gauche*(+) rotamer structure, there is an hydrogen-bond formation between the NAYA amide group (donor) and the NAYA acetyl carbonyl (acceptor) that leaves the NAYA tyrosyl group far apart from these latter groups being mostly exposed and to interact with solvent molecules, as already mentioned. Therefore, a significant concentration of protons (solutions at pH 3 and pH 2) is required to disrupt the above referred hydrogen-bonded complex corresponding to the locked NAYA *gauche*(+) rotamer structure. It has been reported that no hydrogen exchange protection or, a very weak protection, occurs in the submillisecond burst phase for proteins [4]. Thus, this information leads to the situation where we can be dealing instead with a significant hydrogen exchange protection in the burst phase and disruption of the referred hydrogen-bonded complex, corresponding to the locked NAYA *gauche*(+) rotamer structure indeed occurs. To this regard, for NAYA solutions at pH 3 and pH 2 the *trans* and *gauche*(−) rotamers, become the most populated in the burst phase. Although we already assumed that burst phase is occurring in the stopped-flow experiments for NAYA, the significant hydrogen exchange protection noted is a further reinforcement that the literature argued solvent-dependent modification criteria in the burst phase appeared to be valid [4].

**Fig. 2:**
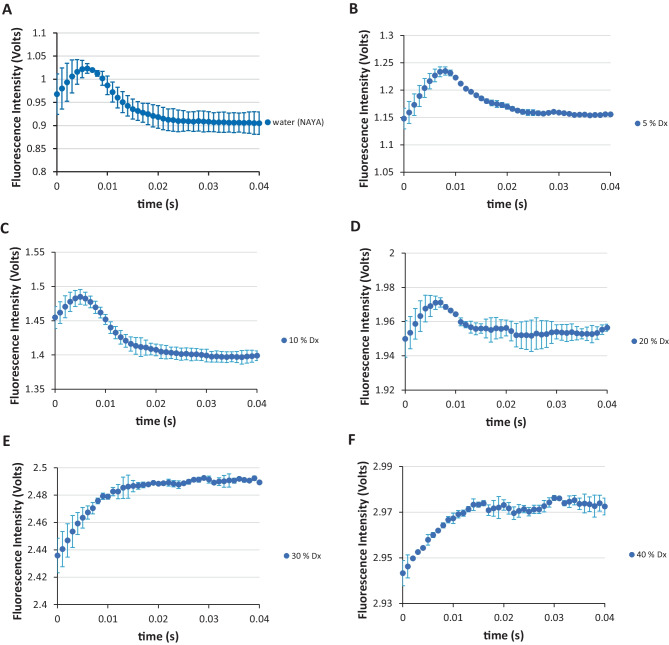
SFM-4 stopped-flow fluorescence intensity measurements in the first 40 milliseconds for NAYA in water and for NAYA aqueous solutions containing 1,4-dioxane (Dx). **A** NAYA in water, **B** NAYA aqueous solution with 5% (v/v) of 1,4-dioxane, **C** NAYA aqueous solution with 10% (v/v) of 1,4-dioxane, **D** NAYA aqueous solution with 20% (v/v) of 1,4-dioxane, **E** NAYA aqueous solution with 30% (v/v) of 1,4-dioxane and **F** NAYA aqueous solution with 40% (v/v) of 1,4-dioxane. In the representations, the average values of the recorded stopped-flow fluorescence intensity are shown and error bars correspond to the standard deviation of three reproducible measurements (*N* = 3 ± std) for each of the two independent experiments performed

We performed further experiments for the NAYA parent compound in order to determine the level of hydrogen exchange protection in the solutions (Fig. 2). In these experiments, we added the aprotic and water miscible 1,4-dioxane solvent to the NAYA aqueous solutions. In Fig. 2A it can be seen for the NAYA compound in water different stopped-flow traces as the ones observed for the NAYA compound in 10 mM tris-HCl at pH 7 (Fig. 1C). While for NAYA in buffer at pH 7 it can be observed a decrease of the recorded fluorescence intensity in the first 3 milliseconds followed an increase of it in the remaining 17 milliseconds of the burst phase (Fig. 1C), for NAYA in water we have the opposite effect (Fig. 2). In this latter case, we have the increased of the recorded fluorescence intensity until ca. 7 milliseconds followed by the decrease of it in the remaining time of the burst phase (see Fig. 2A, for example). Although the results are opposed, this is due to the higher turbulence regime of NAYA in water (devoid of buffer species) in comparison to that for NAYA in buffer and also to the fact that for NAYA in water there could be an increased exposure of the NAYA tyrosyl group, which recalls to an increased population of the NAYA *gauche*(+) rotamer with respect to the NAYA *gauche*(−) and *trans* rotamers. In fact, the referred increased fluorescence intensity in the first ca. 7 milliseconds reports for the fully exposure of the NAYA tyrosyl group in a higher turbulence regime, where signal instability can occur and after that time progressive fluorescence intensity quenching of the exposed NAYA tyrosyl group occurs (see Fig. 2A, for example). We retrieve that for NAYA in water we have capture mostly the *gauche*(+) rotamer population, while for NAYA in buffer the three rotamers mainly co-exist in the burst phase time.

With respect to the effect of adding the aprotic 1,4-dioxane solvent to NAYA in water (Fig. 2), only for a content ≥ 30% (v/v) of 1,4-dioxane an alteration in the stopped-flow traces occurred. Meaning that the closed shape conformation involving an intramolecular hydrogen-bond between the NAYA amide group and the NAYA acetyl group (carbonyl) [17] is indeed reporting to a significant proton exchange protection.

**Fig. 3:**
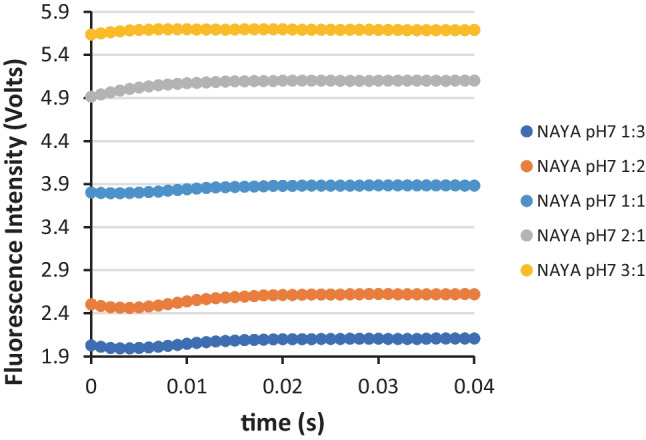
SFM-4 stopped-flow fluorescence intensity measurements for the NAYA compound at pH 7. In the latter representation, it is exhibited the fluorescence intensity traces for different volume proportions of the starting solutions. In this representation, the average values of the recorded stopped-flow fluorescence intensity are shown and error bars (enclosed in the data points) correspond to the standard deviation of three reproducible measurements (*N* = 3 ± std) for each of the two independent experiments performed

We have here considered that the turbulence resulting from the rapid mixing of the starting solutions progressively decrease with the time of the burst phase. In this context, we decided to explore the referred turbulence effect by the rapid mixing of the starting solutions in several volume proportions, namely of 1:3, 1:2, 1:1, 2:1 and 3:1 (Fig. 3). In the referred volume proportions, the first position retrieves to the NAYA buffered solution at pH 7 (10 mM tris-HCl) and the second position refers to the same buffer solution but in which the NAYA compound is absent. A turbulence decrease of the rapid mixed starting solutions is expected to occur in the following order: 3:1 > 2:1 > 1:1 > 1:2 > 1:3, i.e. when the volume proportion of the starting solution containing the NAYA compound in buffer increases. Concomitant with this, is the increase of the NAYA concentration in the resulting rapid mixed starting solutions. In Fig. 3 at a first glance the increase of the recorded overall fluorescence intensity as the concentration of NAYA compound in buffer increases can be seen, as expected. But a close inspection of Fig. 3 reveals differences in the stopped-flow traces within the first 20 milliseconds, i.e. in the burst phase. Still in Fig. 3, for an increased turbulence of the mixed starting solutions, i.e. for the volume proportions of 1:3, 1:2 and 1:1, there is, in the burst phase, the already mentioned decrease of the fluorescence intensity in the first few milliseconds followed by the increase of the fluorescence intensity for the remaining milliseconds. It should be mentioned that for the volume proportions of 2:1 and 3:1, there is, in the burst phase, the increase of the fluorescence intensity only. Meaning that, when the turbulence of the mixed starting solutions decreases a less exposure of the NAYA hydrophobic tyrosyl group potentially occurs, as expected, leading to the abolishment of the already mentioned decrease of the collected fluorescence intensity in the first few milliseconds.

### Burst Phase Analysis for the Ubiquitin and for the Syn Pproteins

**Fig. 4:**
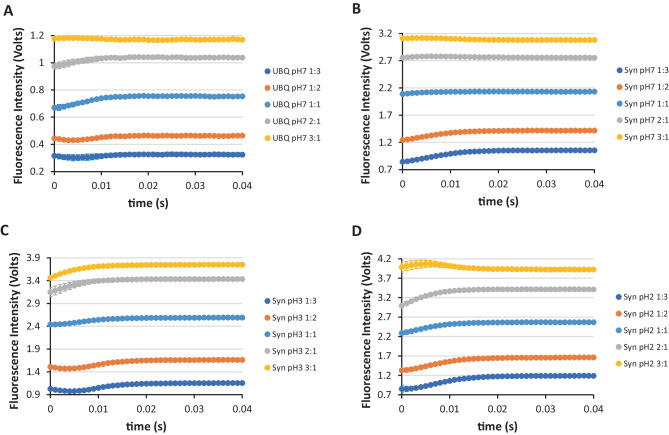
SFM-4 stopped-flow fluorescence intensity traces for the first 40 milliseconds. **A** UBQ protein at pH 7, **B** Syn protein at pH 7, **C** Syn protein at pH 3 and **D** Syn protein at pH 2. In each representation, the fluorescence intensity traces for different volume proportions of the starting solutions are exhibited. In these representations, the average values of the recorded stopped-flow fluorescence intensity are shown and error bars (mostly enclosed in the data points) correspond to the standard deviation of three reproducible measurements (*N* = 3 ± std) for each of the two independent experiments performed

With what has been above described for the NAYA compound and its burst phase analysis, we are in a position to present the results obtained by stopped-flow spectrometry for more complex molecular structures, such as proteins. Before presenting the results obtained for the Syn amyloid protein, we decided to investigate the ubiquitin (UBQ) protein by stopped-flow spectrometry and its burst phase occurrence. It is advantageous to proceed in this way as UBQ is a small globular protein with 76 amino acid residues (8.5 kDa) and contains one tyrosine residue (Y59) (no tryptophan residues, no cysteine residues, or disulphide bridges). As already mentioned, NAYA is an adequate model compound of a tyrosine within a protein. In Fig. 4A, the stopped-flow fluorescence intensity traces obtained for UBQ at pH 7 (10 mM tris-HCl) with varying the volume proportions of the starting solutions in its event of rapid mixing is shown, as similarly described in Fig. 3, for the NAYA parent compound. The final UBQ concentration was 144 µM (*A*_275 nm_ = 0.2; ε = 1390 M^− 1^ cm^− 1^). Comparing Fig. 3, for the NAYA parent compound, with Fig. 4A, for the UBQ protein, there are some resemblances in the burst phase. For the higher turbulence regime of the mixed starting solutions for UBQ, i.e. concerning the volume proportions of 1:3 and 1:2, we have the very initial decrease of the UBQ tyrosyl group fluorescence intensity (Fig. 4A) followed by the increase of the UBQ tyrosyl group fluorescence intensity for the remaining time of the burst phase, as similarly observed for the NAYA parent compound (Fig. 3). For the volume proportions of 1:1 and 2:1, which corresponds to a less turbulence of the mixed starting solutions for UBQ, we observed an increase of the recorded UBQ tyrosyl group fluorescence intensity in the burst phase (Fig. 4A). Surprisingly, for the less turbulent rapid mixed starting solutions, corresponding to the volume proportion of 3:1, we noted an initial increase of the UBQ tyrosyl group fluorescence intensity in the burst phase followed by a decrease of its fluorescence intensity into the stationary phase (Fig. 4A). This is itself an indication that there is no hydrogen exchange protection reported to occur for UBQ, and the possible disruption of the hydrogen-bonded complex formed of nearby aspartates or glutamates protein residues with the UBQ tyrosyl group in the burst phase. Therefore, it seems that burst phase analysis for UBQ retrieves important information regarding the eventual enrolment of a solvent exposed protein Y59 and the stability of several hydrogen-bonded complexes being formed with the UBQ tyrosyl group in the turbulence regimes of the rapid mixed starting solutions in the burst phase.

With the results described for the monomeric UBQ small protein containing a single and exposed tyrosine residue (Y59), we are in a better position to present the results obtained for the Syn amyloid protein and to describe its burst phase behaviour under stopped-flow spectrometry. The Syn amyloid protein with its propensity to aggregate in solution is in similarity to UBQ, a small but disordered protein, containing 140 amino acid residues (14.5 kDa). In the Syn molecular structure, there are four tyrosine residues (Y39, Y125, Y133 and Y136). One of the tyrosine residues (Y39) is located in the N-terminus and the other three tyrosine residues (Y125, Y133 and Y136) are located at the C-terminus. In Fig. 4B the stopped-flow fluorescence intensity traces obtained for a final 33.5 µM protein concentration (*A*_275_ nm = 0.2; ε = 5974 M^− 1^ cm^− 1^) (10 mM tris-HCl, pH 7) in the first 40 milliseconds with varying the volume proportions of the starting solutions is shown, as similarly described for the NAYA parent compound (Fig. 3) and for the UBQ protein (Fig. 4A). The optical density of the Syn protein matches those used for the NAYA parent compound and for the UBQ protein. The Syn protein at pH 7 under stopped-flow spectrometry conditions revealed, as similarly encountered for the NAYA parent compound and for the UBQ protein, a 20 milliseconds time region where the recorded fluorescence intensity varies, designated as the burst phase. But in the burst phase, the Syn protein at pH 7, while under the different regimes of turbulence of the rapid mixed starting solutions (i.e. the different volume proportion of the starting solutions), mostly the increase of the fluorescence intensity is observed (Fig. 4B). This behaviour is distinct from the ones observed for the NAYA parent compound and for the monomeric UBQ protein investigated, where in particular for the latter, for higher turbulence of the rapid mixed starting solutions (i.e. especially for the volume proportions 1:3 and 1:2), there was initially a decrease of the collected fluorescence intensity followed by an increase of the fluorescence intensity in the burst phase (Figs. 3 and 4A). It can be said that such an increase of the collected fluorescence intensity in the burst phase for Syn at pH 7 retrieves to the fact that the Syn tyrosyl groups are not significantly solvent exposed, in particular regarding the higher turbulence regimes of the rapid mixed starting solutions. Therefore, it can be said that the Syn amyloid protein could not be in the monomeric state being eventually aggregated at pH 7, as it is revealed by its burst phase analysis. We previously reported that the increased population of the *trans* rotamer, in particular, is observed for the Syn protein when its aggregation occurs [11, 13]. These studies we conducted for Syn in water in the presence of the 1,4-dioxane [11] and for the Syn protein at pH 7 while increasing the temperature of the amyloid protein solutions [13]. Meaning in the light of the above mentioned the population of the *trans* rotamer is significant enhanced for Syn at pH 7 in the burst phase time.

Concerning to the Syn protein at pH 3 (Fig. 4C), the stopped-flow traces in the burst phase are different from those presented for this amyloid protein at pH 7 (Fig. 4B). In the former case, there is initially the decrease of the protein tyrosyl groups fluorescence intensity, followed by the increase of the protein tyrosyl groups fluorescence intensity in the burst phase, especially for higher turbulence of the rapid mixed starting solutions (Fig. 4C). This scenario is somehow similar to the one observed for the monomeric folded UBQ protein (Fig. 4A). It can be said that for Syn at pH 3, in the burst phase, appears to be monomeric, contrarily to what was proposed to occur for Syn at pH 7, under the same conditions. The explanation for this is provided by the fact that there is no abolishment of the *gauche*(+) rotamer in Fig. 4C, particularly for the higher turbulence regime of the burst phase. This was also observed for the ubiquitin monomeric protein in the higher turbulence regime of the burst phase, as it can be seen in Fig. 4A. Meaning in the light of the already mentioned the population of the *trans* rotamer is not significant enhanced for Syn at pH 3 in the burst phase time. Nevertheless, it dubious the fact that Syn is mostly in a monomeric state since it is well known in the literature that the almost neutralized C-terminus at pH 3 (partial protonation of aspartate and glutamate protein residues) is in contact with the hydrophobic NAC domain in the so-called hydrophobic collapse [18, 19]. In this case, whether the three tyrosine residues in the C-terminus are or not involved in such hydrophobic collapse, the amyloid protein at pH 3 has certainly an increase hydrophobic surface which could increase its propension to aggregate.

We also decided to investigate the Syn protein at pH 2 and the stopped-flow fluorescence intensity traces corresponding to the burst phase were different (Fig. 4D) from those of the Syn protein at pH 3 (Fig. 4C) but, however, comparable with those of the Syn protein at pH 7 (Fig. 4B). This signifies that the Syn protein at pH 2 is likely to be also aggregated. The increase of the turbulence regime, such as with the decrease of the Syn protein volume proportion in the rapid mixed starting solutions, revealed an increase of the fluorescence intensity in the burst phase which is concomitant with the Syn protein to be also aggregated at pH 2. Within a lower turbulence regime for Syn (i.e. in the volume proportion of 3:1) it appears that the Syn protein fluorescence intensity initially increases followed by a decrease of it in the burst phase (Fig. 4D).

**Fig. 5:**
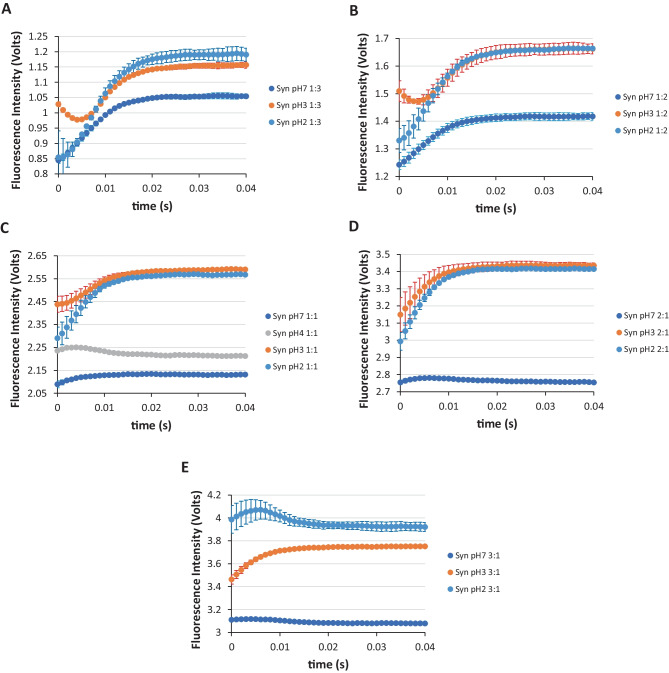
SFM-4 stopped-flow fluorescence intensity traces for the first 40 milliseconds by using different volume proportions of the starting solutions: **A** 1:3, **B** 1:2, **C** 1:1, **D** 2:1 and **E** 3:1. In each representation, the fluorescence intensity traces for the Syn protein at different solution pH are exhibited. The majority of the fluorescence intensity traces here presented are from Fig. 4B, D

In Fig. 5A, E, we have further assembled the results presented in Fig. 4B, D, especially concerning the different turbulence regimes for the Syn protein, i.e. by highlighting the stopped-flow fluorescence intensity curves for the same volume proportion of the rapid mixed starting solutions. It seems from Fig. 5 for the Syn protein at pH 7 that the increase of the fluorescence intensity in the burst phase retrieves to the fact that the protein is aggregating. A close inspection of Fig. 5D, E reveals an initial increase of the Syn protein at pH 7 fluorescence intensity followed by a decrease of it in the remaining time of the burst phase. The occurrence of such an effect retrieves to the less turbulence regime for the Syn protein at pH 7, as similarly observed for the UBQ protein at pH 7 (Fig. 4A). This effect is explained on the basis that the formation of hydrogen-bonded complexes between the Syn tyrosine residues and nearby aspartate and glutamate protein residues at solution pH 7 occurs, which is expected in the steady-state. Furthermore, in this lower turbulence regime of the burst phase and particularly in the beginning of the burst phase the fluorescence intensity increases which means the complexes are disrupted. They only appear at the end of the burst phase time concomitant with the decrease of the fluorescence intensity (Fig. 4A, B, D). This decrease of the fluorescence intensity at the end of the burst phase time is due to the increase formation of the referred hydrogen-bonded complexes where charge transfer occurs. Such an effect was not observed for the NAYA parent compound (Fig. 3). Previously, it was inferred that the Syn protein at pH 3 was in a monomeric state [5] in the burst phase according to the analysis performed. In the high turbulence regime for the Syn protein, i.e. in the volume proportions of 1:3, 1:2 and 1:1 of the starting solutions, the very initial decrease of the Syn protein fluorescence intensity indicates that the protein tyrosyl groups are likely solvent exposed due to the progressive quenching of the Syn tyrosyl groups fluorescence emission with the time of the burst phase (Fig. 5A, C). In addition, the increase of the Syn protein fluorescence intensity is justified by the above mentioned increase of the population of the *trans* and *gauche*(−) rotamers, as referred earlier for the NAYA parent compound and for the ubiquitin protein. What seems to occur is that the Syn tyrosyl groups becomes initially exposed to solvent and then a conformation alteration occurs in the millisecond time that retrieves the Syn tyrosyl groups to a more hydrophobic environment with the time of the burst phase. Meaning that, we are in position to assume that a very likely conformation alteration conferring to the hydrophobic collapse of the Syn protein at pH 3 is occurring in the millisecond burst phase. And, there is first expansion of the Syn protein at pH 3 with some or all Syn protein tyrosyl groups, being exposed to solvent molecules (decrease of the Syn protein fluorescence intensity) and then the contraction of this protein occurs with these Syn tyrosyl groups sensing a more hydrophobic environment (increase of the Syn protein fluorescence intensity). In Fig. 5C, we have the stopped-flow fluorescence intensity trace for the Syn protein at pH 4 and it reveals a very initial increase of the Syn protein fluorescence intensity followed by a decrease of it in the burst phase. This situation resembles the one observed for the Syn protein at pH 2 (Fig. 5E). Although, the arguments are different for the present case, since this amyloid protein at pH 4 possesses at net charge close to zero (Syn pI = 4.7). The tyrosyl group is mostly hydrophobic. When protein solutions pH is at their isoelectric point (pI) the surface net charge is zero, but that does not mean that ionic interactions are not present even in the globular proteins surface. For a disordered protein, such as Syn, there must be an increase of ionic interactions in the protein surface at the pI, in comparison to those in globular proteins. Therefore, the formation of hydrogen-bonded complexes formed between the protein tyrosyl groups with nearby aspartate and glutamate anionic residues could be a simple way to reduce the number of ionic interactions in the Syn protein surface. Additionally, the decrease of the fluorescence intensity for Syn at pH 4 (Fig. 5C) in the end of the burst phase resembles the lower turbulence regime conditions, in which hydrogen-bonded complexes between the protein tyrosyl groups with nearby aspartate and glutamate residues can be formed. It seems within the above mentioned that the Syn tyrosyl groups could be in the Syn protein surface and in a complex form at solution pH 4.

**Fig. 6:**
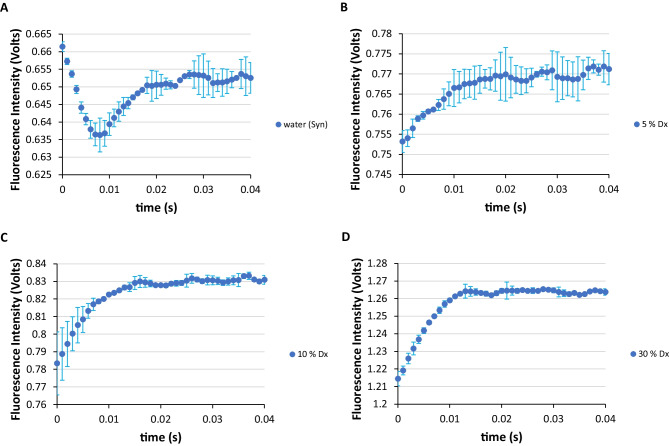
SFM-4 stopped-flow fluorescence intensity measurements in the first 40 milliseconds for Syn in water and for Syn aqueous solutions containing 1,4-dioxane (Dx). **A** Syn in water, **B** Syn aqueous solution with 5% (v/v) of 1,4-dioxane, **C** Syn aqueous solution with 10% (v/v) of 1,4-dioxane and **D** Syn aqueous solution with 30% (v/v) of 1,4-dioxane. In the representations, the average values of the recorded stopped-flow fluorescence intensity are shown and error bars correspond to the standard deviation of three reproducible measurements (*N* = 3 ± std) for each of the two independent experiments performed

We also performed experiments for the Syn amyloid protein in order to determine the level of hydrogen exchange protection in the protein solutions (Fig. 6). In these experiments, we added the aprotic and water miscible 1,4-dioxane solvent to Syn aqueous solutions. For Syn in water it requires only a content of 5% (v/v) of 1,4-dioxane to an alteration in the stopped-flow traces to occur (Fig. 6). The literature reported very weak proton exchange protection for proteins is indeed a fact [4].

**Fig. 7:**
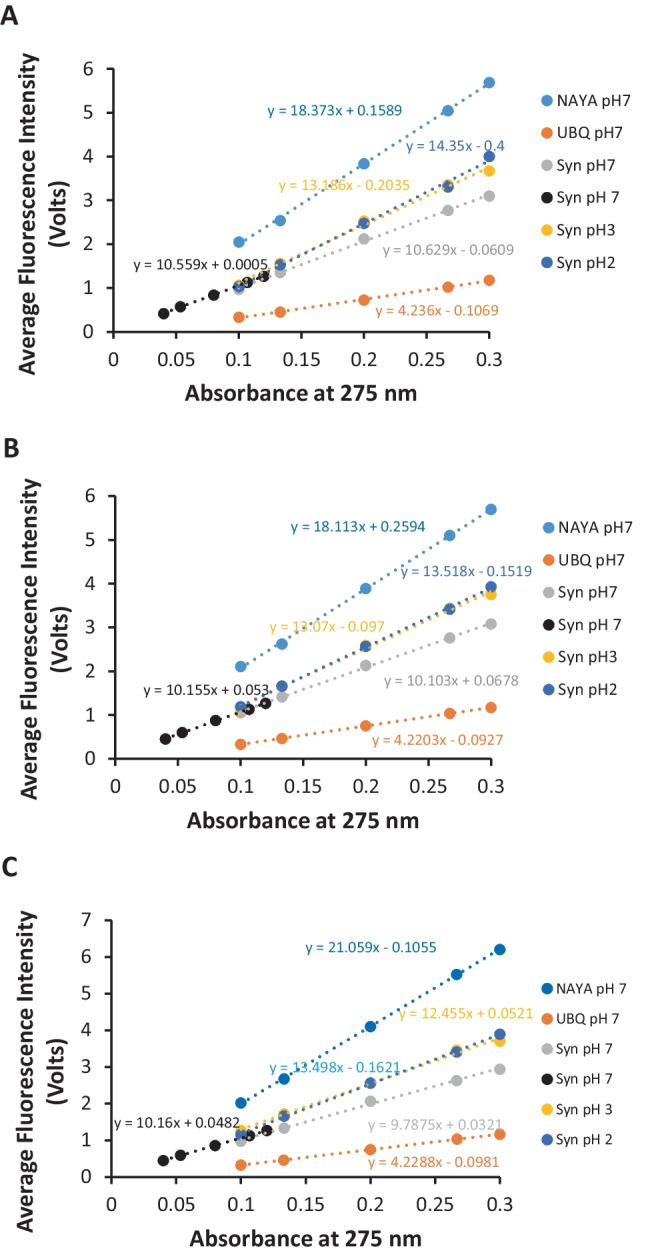
Average values of the recorded fluorescence intensities determined by stopped-flow spectrometry as a function of the molecular species absorbance at 275 nm. **A** First 20 milliseconds, **B** subsequent 20 milliseconds and **C** 8 s (excluding the first 40 milliseconds). In these representations, it is exhibited the average values of the recorded fluorescence intensities for the molecular species investigated in this study, namely NAYA at pH 7, UBQ at pH 7, Syn at pH 7, Syn at pH 3 and Syn at pH 2. Matched optical densities of the referred molecular species at pH 7 were used when adequate

We further decided to determine if Syn protein aggregation is indeed occurring in the burst phase in comparison to the subsequent steady-state conditions. To this regard, we firstly determined the average values of the recorded stopped-flow fluorescence intensities for the molecular species used in this study (the NAYA parent compound, the UBQ protein and the Syn amyloid protein), i.e. concerning the fluorescence intensities determined in the burst phase, corresponding to the first 20 milliseconds, to the subsequent 20 milliseconds of the burst phase and even to the subsequent 8 s (excluding the first 40 milliseconds). Therefore, these determined average values of the recorded fluorescence intensities for the molecular species investigated in this study were represented as a function of the molecular species concentration, as shown in Fig. 7. It can be seen that linear trend lines were thus obtained (Fig. 7). We also performed stopped-flow experiments, particularly with a lower Syn concentration, of 13.4 µM (*A*_275 nm_ = 0.08, ε = 5974 M^− 1^ cm^− 1^). It can be seen in Fig. 7 that linear trends are also obtained for both the higher (33.5 µM) and the lower (13.4 µM) Syn concentrations tested. In the first 20 milliseconds, the slope for the lower Syn concentration is inferior in comparison with the slope obtained for the higher Syn concentration investigated (Fig. 7). This is to be expected since less aggregation or even no aggregation is to be expected for the lower Syn concentration studied. Still referring to the slopes retrieved from the lower and the higher Syn concentrations studied, for the 20–40 milliseconds and for first 8 s (excluding the first 40 milliseconds) the slopes for the lower Syn concentration studied are higher than the slopes for the higher Syn concentration investigated (Fig. 7B, C). It can be deduced that the latter slopes follow the same tendency and static conditions of the system arise after the first recorded 20 milliseconds. To reinforce, the slopes for the lower Syn concentration tested, where minor aggregation or even no aggregation is to be expected, are the same (Fig. 7B, C).

### Identification of the Syn Protein Collapse and of Its Expansion at pH 3 in the Burst Phase

**Fig. 8:**
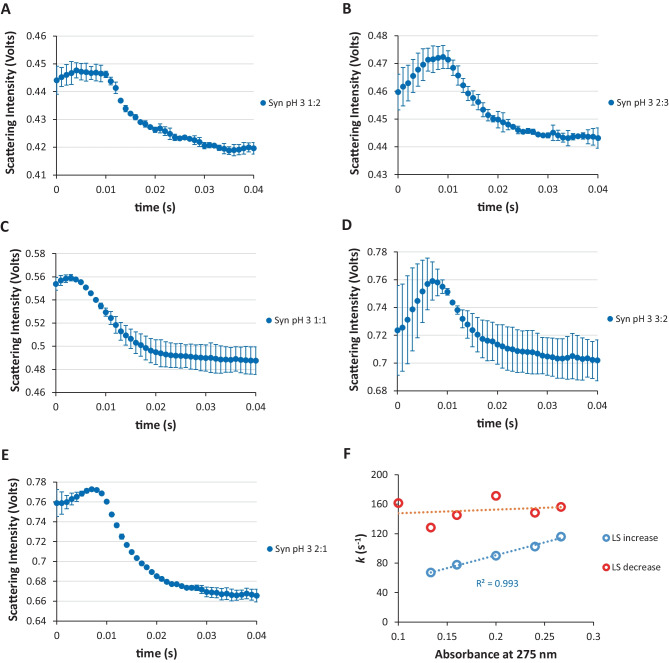
SFM-4 stopped-flow light scattering (LS) traces as a function of time (first 40 milliseconds) (λ_excitation_ = 378 nm and λ_emission_ > 290 nm). Protein:buffer ratio **A** 1:2, **B** 2:3, **C** 1:1, **D** 3:2 and **E** 2:1. **F** Calculated first order rate constants for Syn solutions at pH 3 as a function of the protein absorbance at 275 nm. LS increase refers to the first ca. 7 milliseconds of the burst phase and LS decrease stands for the subsequent ca. 7 milliseconds of the burst phase. The data in this figure is from two independent experiments, i.e. the data was recorded in different days. Error bars correspond to the standard deviation of three reproducible measurements (*N* = 3 ± std) for each independent experiment

The ANS fluorescent dye seems to provide information for the existence of a molten globule state for globular proteins in the process of folding [20–22]. Nevertheless, we thought about another strategy that could be more adequate, i.e. the identification of both the collapse of the Syn and its precedent protein expansion at solution pH 3. To this regard, we decide to monitor the light scattering in the millisecond time of the burst phase under stopped-flow spectrometry conditions. Therefore, in Fig. 8A, E we show the recorded light scattering signal with the time of the burst phase for Syn protein solutions at pH 3 while varying the protein concentration. It can be seen from this figure that there is an initial increase of the light scattering signal in the first ca. 7 milliseconds and, following this, a decrease of the light scattering signal can be observed. The mentioned initial increase of the light scattering signal could indicate that the protein scatters light less efficiently due to the protein expansion at pH 3 and then reaches a maximum of the recorded light scattering at ca. 7 milliseconds which can be also due to the protein collapse at pH 3, by scattering light more efficiently. In order to be assured that the referred initial increase of the light scattering up to ca. 7 milliseconds corresponds to Syn in a monomeric state at pH 3 we determined the first order rate constants and in fact these rate constants follow a linear trend with the protein monomeric concentration at pH 3 (Fig. 8F). Meaning, that we are confident that the processes retrieving to the first ca. 7 milliseconds are concerned with the protein being in a monomeric state at pH 3. After the ca. 7 milliseconds, the determined first order rate constants do not depend on the monomeric protein concentration at pH 3 (Fig. 8F). This reinforces the fact that the decrease of the light scattering signal after the ca. 7 milliseconds of the burst phase is due to the start of the protein aggregation. This also reinforces the fact that the protein collapse at ca. 7 milliseconds triggers the consequent events of the protein aggregation.

We must emphaticise that the burst phase under stopped-flow spectrometry conditions has unique features due to the existent turbulence regime and can reveal newly and unexplored interactions for amyloid protein systems. This is different from stationary conditions and even the burst phase does not retrieve solely to very fast events occurring in these systems. Rather, we can say that in the burst phase we are studying the molecular events under non-equilibrium conditions.

## Discussion

In this report, we decided to perform the burst phase analysis for the disordered Syn amyloid protein. This is advantageous since the burst phase analysis has been used for the identification of folding intermediates present in the refolding experiments of several unfolded proteins. But the identification of these folding intermediates in the refolding experiments has been severely questioned in the literature [3, 4], in which some studies argued that a particular solvent-dependent modification is occurring in the still unfolded polypeptide chain [4]. These claims seemed reasonable if we consider the real complexity of the processes occurring in the rapid mixed starting solutions in the burst phase. In the burst phase the molecular systems are not static and they are dependent of the micromixing effects until the rapid mixture of the starting solutions eventually stops and enters in the stationary phase. One aspect is characterization of the processes underlying micromixing in stopped-flow spectrometry and the other aspect is the optical characterization of the molecular systems undergoing micromixing in stopped-flow spectrometry. If the first aspect is not fully understood, as it is very challenging, difficulties arise to the further characterization of the molecular systems under stopped-flow spectrometry conditions. But what is attractive in the burst phase is the event of the newly collapse phase that can retrieve valid information and the disclosure of newly unexplored interactions for the intervenient molecular systems. Although it is difficult to characterize these possible newly unexplored interactions since the event of micromixing is itself complex, a possible strategy is to use different molecular systems, of different complexity, in order to understand the simpler solvent interaction effects and next going to more complex molecular interactions. This, in fact, was indeed pursued in the current study, as we firstly investigated the NAYA parent small compound in the burst phase, in which no particular reaction is to be expected for this compound, besides solvent interaction. Next, we used the small UBQ protein that has no propensity to aggregate and that remains in a monomeric state. Further, we proceeded to a more complexed molecular system, which involves particular intermolecular interactions leading to the Syn protein aggregation. With respect to the NAYA parent compound, the likely solvent exposure of the NAYA hydrophobic tyrosyl group in the burst phase led us to pursue with the NAYA rotameric conformations in order to describe its behavior in the referred collapse phase. Therefore, the very initial decrease of the NAYA tyrosyl group fluorescence intensity affords for the increased population of the NAYA *gauche*(+) rotamer in a locked conformation. This *gauche*(+) rotamer in a locked conformation has been proposed to occur in non-polar solvents [17] leading to high exposure of the NAYA hydrophobic tyrosyl group. Followed by the very initial decrease of the fluorescence intensity of the NAYA solvent exposed tyrosyl group, which affords for the NAYA tyrosyl group progressive quenching of its fluorescence intensity as the turbulence of the rapid mixed starting solutions becomes minor with the time of the burst phase, we have the rise of the NAYA tyrosyl group fluorescence intensity due the increased population of the *trans* and *gauche*(−) rotamers. In this case, these rotamers experiment the hydrophobic interactions of the NAYA carbonyl groups with the NAYA tyrosyl group leading to the referred increment NAYA fluorescence intensity in the burst phase. Within the above described, we have assigned the contribution of the NAYA rotameric conformations in the interpretation of the NAYA tyrosyl group fluorescence intensities variation in the burst phase. This is mostly the occurrence of solvent-dependent modification and also the particular intramolecular interactions among less solvent exposed NAYA carbonyls groups with the NAYA tyrosyl group. With lowering the pH of the NAYA rapid mixed starting solutions in the burst phase, differences were also encountered, namely the abolishment of the very initial decrease of the NAYA tyrosyl group fluorescence intensity. It requires, therefore, significant amount of protons (pH 3 and pH 2) in order to disrupt the locked *gauche*(+) rotamer, leading to the *trans* and *gauche*(−) rotamers as being the most populated with the time of the burst phase. The use of the NAYA parent small compound allowed us to implement the classic NAYA rotameric conformations as a strategy to interpret the occurrence of solvent-dependent modifications of this solvent exposed small molecule. With regard to the folded UBQ protein, we inferred that it is in a monomeric state and the solvent exposed to single tyrosine residue (Y59) retrieved the discussion made for the NAYA parent compound. No particular molecular interactions are to be expected in the monomeric UBQ protein besides solvent-dependent modification of its single and solvent exposed tyrosine residue. With this information at hand, we are in a position to interpret the more complex molecular system referring to the Syn amyloid protein. In this case, the classic rotameric conformations retrieved for the Syn tyrosyl groups an increase of the population of the *trans* rotamer, which was previously reported to be related with the occurrence of Syn protein aggregation [11, 13]. Therefore, the Syn protein appears to aggregate in the burst phase for pH 7 and pH 2, in particular. For the Syn protein at pH 3, which appears to be in a monomeric state, the classic rotameric conformations retrieving for the Syn tyrosyl groups can in part be applied and it was for the first time captured a molecular conformation alteration leading to the formation of a possible folded intermediate in the milliseconds time scale of the burst phase. Particularly, there is an initial expansion of Syn protein at pH 3 since it is not expected that some or all the Syn tyrosyl groups that in the protein hydrophobic collapse could be exposed to water (decrease of the protein fluorescence intensity). And, then the Syn protein at pH 3 contracts with its tyrosyl groups sensing a more hydrophobic environment (increase of the protein fluorescence intensity). One aspect that deserves mentioning is the identification of hydrogen-bonded complexes formation with protein tyrosyl groups and nearby aspartate and glutamate residues in the lower investigated turbulence regimes for the UBQ protein at pH 7 and for the Syn protein at pH 7.

## Conclusions

Due to the challenge in interpreting the collapse phase occurring in the event of micromixing under stopped-flow spectrometry, we designed experiments by increasing the complexity of the molecular systems in order to identify and to characterize the molecular interactions involved for the selected molecular species. For the simpler NAYA parent compound and for the monomeric folded UBQ protein the identification and characterization of the molecular interactions involved on basis of the classic rotameric conformations for the NAYA parent compound and for the UBQ protein tyrosine residue while in the burst phase was possible. Although in these latter cases we are dealing with solvent and intramolecular interactions, we decided to further investigate a more complex molecular system which is the disordered Syn amyloid protein. The results obtained were challenging in their interpretation and in the light that not only solvent and intramolecular interactions can be involved but also intermolecular interactions are involved in the aggregation of the Syn protein. Syn protein aggregation was predicted to occurs at pH 7 and pH 2 in the burst phase, in particular. For the Syn protein at pH 3, it was concluded that the protein is in the monomeric state and a conformation alteration corresponding to the formation of a possible folded intermediate was for the first time proposed to occur in the millisecond time scale of the burst phase. We also want to emphaticise that the study design here adopted helped us to better understand the collapsed phase under stopped-flow spectrometry conditions, despite solvent-dependent modification effects in the disordered Syn amyloid protein, still need further investigation. Considering both the literature criteria, namely the solvent-dependent modification of this disordered amyloid protein as well as the formation of a possible folded intermediate by fast contraction of the firstly expanded disordered protein, are likely to occur in the burst phase [3, 4].

### Supplementary Information

Below is the link to the electronic supplementary material.

Supplementary file1 (DOCX 248 KB)

## Data Availability

All data analysed during this work are included in the submitted manuscript.
