# Supplementary material for: Burst Phase Analysis of the Aggregation Prone α-synuclein Amyloid Protein
Source: J Fluoresc. 2023 Jun 5;34(1):381–95. doi: 10.1007/s10895-023-03285-1 (PMC10808200; doi:10.1007/s10895-023-03285-1)
Supplement: Supplementary file 1 — Supplementary file1 (DOCX 248 KB) [file 10895_2023_3285_MOESM1_ESM.docx]

Burst phase analysis of the aggregation prone α-synuclein amyloid protein

Marco A. Saraiva ^*^ and M. Helena Florêncio

*Correspondence to: Marco A. Saraiva, Av. Rovisco Pais, Instituto Superior Técnico, University of Lisbon, Campus Alameda, 1049-001 Lisbon, Portugal.

[marco.saraiva@tecnico.ulisboa.pt](mailto:marco.saraiva@tecnico.ulisboa.pt)

**Supporting Material**

**SI. 1 SDS-PAGE of the α-synuclein purification**

**SI. 2 Size exclusion chromatography (SEC) of α-synuclein**

**SI. 1 *SDS-PAGE of the α-synuclein purification***

The corresponding SDS-PAGE of the α-synuclein purification is presented in Fig. SI 1. The protein is almost pure after anion-exchange chromatography revealing mostly a band at *ca*. 18 kDa in the SDS-PAGE, which corresponds to α-synuclein (Fig. SI 1a). In order to remove high molecular weight aggregates of α-synuclein, the resulting pooled fractions of anion-exchange chromatography were submitted to size exclusion chromatography. The protein is pure after size exclusion chromatography, revealing a band in the SDS-PAGE at *ca*. 18 kDa (Fig. SI 1b).


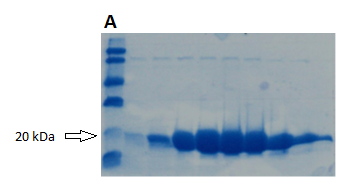


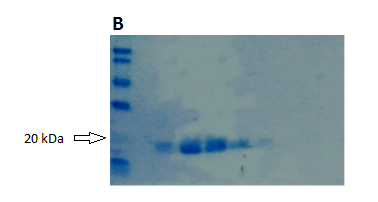


**Figure SI 1.** Some of the collected fractions were applied to a SDS-PAGE (13 % acrylamide) (denaturing conditions) (a) from anion-exchange chromatography and (b) from size exclusion chromatography. α-Synuclein appeared as a band at *ca*. 18 kDa.

**SI. 2 *Size exclusion chromatography (SEC) of α-synuclein***

In Fig. SI 2, the blue line represents the recorded absorbance at 280 nm and the red line represents the recorded conductivity. Absorbance measurements at 280 nm reveal three peaks; the first peak at ca. 43 mL of the elution corresponds to α-synuclein aggregates (as observed in the SDS-PAGE in Fig. SI 1a), the second peak at 60-80 mL of the elution corresponds to pure α-synuclein (as observed in the SDS-PAGE in Fig. SI 1b, and these fractions were pooled, concentrated and used in the biophysical studies, i.e. after dialysis against water overnight) and the second peak at 80-110 mL of the elution corresponds to a low molecular weight impurity that was not detected in the SDS-PAGE in Figs. SI 1a and 1b. Conductivity measurements reveal one peak at 95-110 mL of the elution, which corresponds to the ion species present in the SEC buffer used (50 mM Tris-HCl + 150 mM NaCl at pH 7.5). According to the conductivity measurements (red line), the maximum of the peak (at ca. 100 mL of the elution) corresponding to the referred ion species present in the SEC buffer used is very close to the maximum of the peak corresponding to the mentioned low molecular weight impurity (at ca. 95 mL of the elution) (blue line). This retrieves that the low molecular weight impurity observed possesses a molecular weight far below the 10 kDa (first bottom mark in the SDS-PAGE in Figs. SI 1a and 1b), and therefore this impurity is indeed not possible to be observed in the SDS-PAGE presented in Figs. SI 1a and 1b.

**Figure SI 2.** Original size exclusion chromatogram obtained of α-synuclein samples, after anion-exchange chromatography.
